# Exposure of the U.S. Population to Bisphenol A and 4-*tertiary*-Octylphenol: 2003–2004

**DOI:** 10.1289/ehp.10753

**Published:** 2007-10-24

**Authors:** Antonia M. Calafat, Xiaoyun Ye, Lee-Yang Wong, John A. Reidy, Larry L. Needham

**Affiliations:** Division of Laboratory Sciences, National Center for Environmental Health, Centers for Disease Control and Prevention, Atlanta, Georgia, USA

**Keywords:** biomarkers, biomonitoring, BPA, exposure, NHANES, tOP, urine

## Abstract

**Background:**

Bisphenol A (BPA) and 4-*tertiary*-octylphenol (tOP) are industrial chemicals used in the manufacture of polycarbonate plastics and epoxy resins (BPA) and nonionic surfactants (tOP). These products are in widespread use in the United States.

**Objectives:**

We aimed to assess exposure to BPA and tOP in the U.S. general population.

**Methods:**

We measured the total (free plus conjugated) urinary concentrations of BPA and tOP in 2,517 participants ≥ 6 years of age in the 2003–2004 National Health and Nutrition Examination Survey using automated solid-phase extraction coupled to isotope dilution–high-performance liquid chromatography–tandem mass spectrometry.

**Results:**

BPA and tOP were detected in 92.6% and 57.4% of the persons, respectively. Least square geometric mean (LSGM) concentrations of BPA were significantly lower in Mexican Americans than in non-Hispanic blacks (*p* = 0.006) and non-Hispanic whites (*p* = 0.007); LSGM concentrations for non-Hispanic blacks and non-Hispanic whites were not statistically different (*p* = 0.21). Females had statistically higher BPA LSGM concentrations than males (*p* = 0.043). Children had higher concentrations than adolescents (*p* < 0.001), who in turn had higher concentrations than adults (*p* = 0.003). LSGM concentrations were lowest for participants in the high household income category (> $45,000/year).

**Conclusions:**

Urine concentrations of total BPA differed by race/ethnicity, age, sex, and household income. These first U.S. population representative concentration data for urinary BPA and tOP should help guide public health research priorities, including studies of exposure pathways, potential health effects, and risk assessment.

Of the more than 2,000 high-production volume chemicals that are manufactured in or imported into the United States in amounts of one million pounds or more per year (U.S. Environmental Protection Agency 2004), many are widely used in consumer products. Among these chemicals are bisphenol A [BPA; 2,2-bis(4-hydroxyphenyl)propane; CAS no. 80-05-7] and 4-*tertiary*-octylphenol [tOP; 4-(1,1,3,3-tetramethylbutyl)phenol; CAS no. 140-66-9]. BPA is used in the manufacture of polycarbonate plastic and epoxy resins, which can be used in impact-resistant safety equipment and baby bottles, as protective coatings inside metal food containers, and as composites and sealants in dentistry [Center for the Evaluation of Risks to Human Reproduction (CERHR) 2007; European Union 2003]. Exposure to BPA is thought to result primarily from ingestion of food containing BPA (Kang et al. 2006; Vandenberg et al. 2007). tOP is both a degradation product of and an intermediate in the manufacture of octylphenol ethoxylates, which are nonionic surfactants used in detergents, pesticide formulations, and other applications (Ying et al. 2002). Exposure to tOP may occur from contact with personal care products, detergents, water, and food containing tOP.

Exposures to tOP can result in developmental and reproductive alterations in aquatic species (Segner et al. 2003) and in laboratory animals (Aydogan and Barlas 2006; Bian et al. 2006; Blake et al. 2004; Nagao et al. 2001; Willoughby et al. 2005). At high doses, BPA demonstrates estrogen-like effects on uterine and prostate organ weights in experimental animals. At doses below the putative lowest observed adverse effect level, exposure to BPA has reportedly resulted in decreased sperm production, increased prostate gland volume, altered development and tissue organization of the mammary gland, altered vaginal morphology and estrous cycles, disruption of sexual differentiation in the brain, and accelerated growth and puberty (Durando et al. 2007; Howdeshell et al. 1999; Kubo et al. 2003; Richter et al. 2007; Rubin et al. 2006; Schonfelder et al. 2002; Timms et al. 2005; vom Saal et al. 1998). At present, the interpretation of the evidence related to the low-dose effects of BPA is a subject of scientific debate (European Union 2003; Goodman et al. 2006; Gray et al. 2004; National Toxicology Program 2001; vom Saal and Hughes 2005).

BPA and tOP are of concern to environmental public health because of the high potential for exposure of humans to these phenols and their demonstrated animal toxicity. Information about the concentrations of these compounds in the general population is important for understanding human exposure to BPA and tOP. The National Health and Nutrition Examination Survey (NHANES), conducted continuously since 1999 by the National Center for Health Statistics of the Centers for Disease Control and Prevention (CDC), is designed to measure the health and nutritional status of the civilian noninstitutionalized U.S. population ≥ 2 months of age (CDC 2003). The surveys include household interviews; collection of medical histories; standardized physical examinations; and collection of biologic specimens (e.g., blood and urine from participants ≥ 1 and ≥ 6 years of age, respectively) for clinical chemistry testing, nutritional indicators assessments, and assessment of exposure to environmental chemicals (CDC 2005, 2006).

Previously, we analyzed 394 urine samples collected from adult participants of NHANES III, conducted during 1988–1994, to estimate urinary concentrations of total BPA (free plus conjugated species) in selected demographic groups (Calafat et al. 2005). We now report the first estimate of urinary concentrations of total BPA and tOP in NHANES 2003–2004 participants, a representative sample of the noninstitutionalized U.S. population ≥ 6 years of age.

## Materials and Methods

NHANES participants are selected based on their age, sex, and racial/ethnic background through a complex statistical process using the most current census information. Persons on full-time active duty with the U.S. armed forces are not eligible to participate (CDC 2006). Urine samples analyzed in this study were obtained from 2,517 people, a one-third random subset of participants in NHANES 2003–2004. The National Centers for Health Statistics Institutional Review Board reviewed and approved the study protocol. Informed written consent was obtained from all participants; parents or guardians provided consent for participants < 18 years of age.

One spot urine sample per participant was collected during one of three daily examination session periods (i.e., morning, afternoon, evening). We measured the total urinary concentrations (free plus conjugated species) of BPA and tOP using online solid-phase extraction (SPE) coupled to high-performance liquid chromatography (HPLC)–isotope dilution tandem mass spectrometry (MS/MS) with peak focusing as described before (Ye et al. 2005). Briefly, the conjugated species of BPA and tOP in 100 μL urine were hydrolyzed by use of β-glucuronidase/sulfatase (*Helix pomatia* H1; Sigma Chemical Co., St. Louis, MO). After hydrolysis, samples were acidified with 0.1 M formic acid; BPA and tOP were preconcentrated by online SPE, separated by reversed-phase HPLC, and detected by atmospheric pressure chemical ionization–MS/MS. The limits of detection (LODs) were 0.4 μg/L (BPA) and 0.2 μg/L (tOP). The LOD, calculated as 3*S*_0_, where *S*_0_ is the standard deviation as the concentration approaches zero, is the concentration at which a measurement has a 95% probability of being greater than zero (Taylor 1987). Depending on the concentration, the coefficients of variation ranged from 11% to 13% for BPA and from 17% to 25% for tOP [Supplemental Material, Table 1 (online at http://www.ehponline.org/members/2007/10753/suppl.pdf)]. Low-concentration (~ 4 μg/L) and high-concentration (~ 20 μg/L) quality control materials, prepared with pooled human urine spiked with the analytes of interest, were analyzed with standard, reagent blank, and NHANES samples.

We performed statistical analyses using SAS (version 9.1.3; SAS Institute Inc., Cary, NC) and SUDAAN (version 9.0.1; RTI International, Research Triangle Park, NC). SUDAAN calculates variance estimates after incorporating the sample population weights, which account for unequal selection probabilities and planned oversampling of certain subgroups resulting from the complex multistage probability design of NHANES. We calculated geometric means (if the overall weighted frequency of detection was > 60%) and distribution percentiles for both volume-based (micrograms per liter) and creatinine-corrected concentrations (micrograms per gram creatinine). For concentrations below the LOD, a value equal to the LOD divided by the square root of 2 was used in the univariate and multivariate analyses (Hornung and Reed 1990). Because the concentrations of BPA and tOP were not normally distributed, we used their natural log transformation.

We used analysis of covariance to examine how well selected variables were associated with the log-transformed urine concentrations of BPA. Age, reported in years at the last birthday, was categorized in four groups (6–11, 12–19, 20–59, and ≥ 60 years). Participants were categorized as smokers if their serum cotinine concentrations were > 10 μg/L. Only 6% of children and adolescents (and nobody < 11 years of age) were considered smokers. On the basis of self-reported data, we categorized race/ethnicity into three groups: non-Hispanic blacks, non-Hispanic whites, and Mexican Americans. Persons not included in one of these three race/ethnicity groups were included only in the total population estimate. Also on the basis of questionnaire responses, annual household income was available in increments of $5,000 (ranging from < $5,000 to > $75,000). To obtain comparable number of participants per income group, we categorized income as < $20,000, $20,000–$45,000, and > $45,000. We considered all possible two-way interactions. For the multiple regression analyses, we calculated the least square geometric mean (LSGM) concentrations of BPA and compared them for each categorical variable. The multiple regression analysis was initially conducted separately for children and adolescents (6–19 years of age) and adults (≥ 20 years of age) with age as continuous variable and smoking status included only for the adult model. We did not include smoking status in the children and adolescents model because of the small proportion and uneven distribution of smokers among these two groups. For children and teens, body mass index (BMI) is both age- and sex-specific, and instead of BMI we used BMI-for-age percentile (BMIPCT), calculated on the basis of a BMI-for-age growth chart for persons 2–19 years of age (Kuczmarski et al. 2002). Because neither BMI nor smoking status in the adult model nor BMIPCT in the model for children and teens were significantly associated with BPA concentrations, these variables were not included in analysis of all ages combined. Because the distribution of creatinine concentrations was skewed, we used their common log transformation.

To arrive at the final model, we used backward elimination with SUDAAN to eliminate the nonsignificant interactions one at a time. Then we removed nonsignificant main effects one at a time and reran the model to determine whether the beta coefficients for significant main effects or interactions changed by > 10%. If any did, we retained the nonsignificant main effect in the model. Once the backward procedure was completed, main effects and interactions were added back into the model one at a time to determine whether any were significant (*p* < 0.05). If any were, they were retained in the final model.

We also compared the geometric mean concentrations of BPA by examination session (i.e., morning, afternoon, evening) for all ages and stratified by age (i.e., children and adolescents, adults).

## Results

BPA was detected in 92.6% of persons ≥ 6 years of age with total concentrations ranging from 0.4 μg/L to 149 μg/L. tOP was detected in 57.4% of samples at total concentrations of 0.2 μg/L–20.6 μg/L (Tables 1 and 2). For BPA, the geometric mean and 95th percentile concentrations were 2.6 μg/L (2.6 μg/g creatinine) and 15.9 μg/L (11.2 μg/g creatinine), respectively. For tOP, the 95th percentile concentration was 2.2 μg/L (2.8 μg/g creatinine); because the overall weighted frequency of detection was < 60%, we did not calculate the geometric mean or conduct multivariate analysis.

In the regression model for BPA for all ages, we included sex, race/ethnicity, age group, household income, and creatinine concentration (Barr et al. 2005) as independent variables. The final model included sex (*p* = 0.04), race/ethnicity (*p* = 0.02), age group (*p* < 0.01), creatinine concentration (*p* < 0.01), and income (*p* = 0.02). The LSGM concentrations (Tables 3 and 4), which provide geometric mean estimates for a demographic variable after adjustment for the model covariates, were significantly lower in Mexican Americans than in non-Hispanic blacks (*p* = 0.006) and non-Hispanic whites (*p* = 0.007); the difference was not statistically significant between non-Hispanic blacks and non-Hispanic whites (*p* = 0.21). Females had statistically higher LSGM concentrations than did males (*p* = 0.043). LSGM concentrations of BPA for those in the low household income category were higher than for those in the high category (*p* = 0.004; Tables 3 and 4). Children had higher LSGM BPA concentrations than adolescents (*p* < 0.001), who in turn had higher concentrations than adults (*p* = 0.003) (Tables 3 and 4).

**Figure 1:**
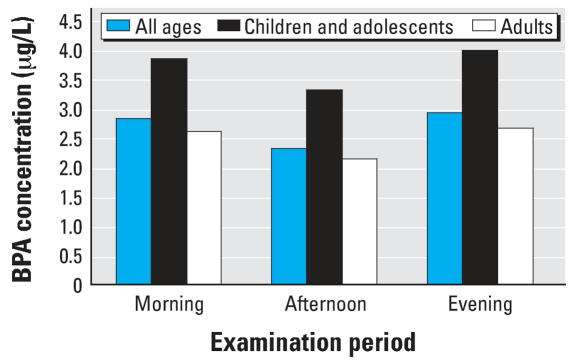
Geometric mean urinary concentrations of BPA (μg/L) for each daily examination period.

We compared the geometric mean BPA concentrations by examination session time, and separately for adults and for children and adolescents (Figure 1). The geometric mean concentrations of BPA were significantly higher in the morning than in the afternoon collections for all ages (*p* = 0.0134) and for adults (*p* = 0.015), but not for children and adolescents (*p* = 0.18). Similarly, the geometric mean concentrations of BPA were significantly higher in the evening than in the afternoon collections for all ages (*p* = 0.0317), but not for children and adolescents (*p* = 0.2), and only marginally different for adults (*p* = 0.06). The geometric mean concentrations of BPA between the morning and evening sessions were not significantly different [Supplemental Material, Table 2 (online at http://www.ehponline.org/members/2007/10753/suppl.pdf)]. When we included examination session time as an independent variable, in addition to sex, race/ethnicity, age group, household income, and creatinine concentration, the final regression model for BPA for all ages, yielded comparable results plus one additional race/ethnicity-by-examination session time interaction term [Supplemental Material, Tables 3–4 (online at http://www.ehponline.org/members/2007/10753/suppl.pdf)].

## Discussion

The free plus conjugated (total) urinary species of BPA were detected in 92.6% of persons ≥ 6 years of age in this sample of the U.S. population. In humans, orally administered BPA is conjugated to the monoglucuronide and excreted (Volkel et al. 2002), with an estimated half-life of BPA of ~ 6 hr (CERHR 2007). Taken together, these data suggest continual exposure to BPA. After human exposure, a fraction of the absorbed BPA may distribute to body storage site(s) (such as adipose tissue) (Fernandez et al. 2007), followed by a slow release into the bloodstream and ultimately into the urine. This would result in a low-dose continuous exposure within the body, similar to that proposed for the insecticide chlorpyrifos (Needham 2005). BPA stored in adipose tissue likely would be in its more lipophilic free form rather than in its hydrophilic conjugates. If the free form is the pharmacologically active species, one question of public health interest is how much of the free BPA is available to interact at the target organ(s). The concentrations of free BPA in circulating blood rather than the total urinary concentrations of BPA would be especially helpful for this assessment. Nevertheless, the NHANES 2003–2004 urinary data suggest that exposure to BPA is prevalent in the U.S. general population and can be used to estimate the distribution of BPA exposures (e.g., using reverse dosimetry) or the daily intake (assuming a steady state excretion). Furthermore, although within-person variability in urinary concentrations of BPA exists (Arakawa et al. 2004; Mahalingaiah et al. 2007; Teitelbaum et al. 2007), concentrations based on one spot sample per person can be useful in calculating mean population concentration estimates in cross-sectional studies (CERHR 2007). Furthermore, data from a recent study including about 80 adults suggest that a single sample is predictive of BPA exposure over weeks to months, and can provide good sensitivity to classify a person’s exposure in epidemiologic studies (Mahalingaiah et al. 2007). Similarly, results from another study conducted among a group of 35 children suggest that BPA concentrations in a single urine sample can be used to categorize the 6-month average exposure to BPA (Teitelbaum et al. 2007).

The total concentrations of tOP were detected in only 57.4% of persons ≥ 6 years of age. BPA concentrations were higher than those for tOP. The concentrations (median, 0.3 μg/L) and frequency of detection of tOP are consistent with previous limited biomonitoring data. In 10 healthy adult (21–28 years of age) Japanese volunteers, the urinary concentrations of tOP were < 0.3 μg/L (Inoue et al. 2003). tOP was measured in five urine and three plasma samples from eight healthy adult (22–25 years of age) Japanese volunteers; tOP concentrations were < 0.02 μg/L (urine) and 0.1–0.2 μg/L (plasma) (Kawaguchi et al. 2004). tOP was detected at concentrations of < 0.05 to 1.15 μg/L in 31 of 180 human cord blood samples collected during delivery at the University Malaya Medical Centre in Malaysia (Tan and Mohd 2003).

The lower frequency of detection of tOP than of BPA in the NHANES 2003–2004 population might be explained by lower exposures to tOP or octylethoxylates (the environmental precursors of tOP) than to BPA and/or by differences in toxicokinetic factors. After oral ingestion, BPA is rapidly metabolized to BPA monoglucuronide and excreted (Pottenger et al. 2000; Volkel et al. 2002). *In vitro* (Pedersen and Hill 2000a, 2000b) and *in vivo* studies in fish (Pedersen and Hill 2002) suggest that metabolism of tOP results in a large number of metabolic products, including oxidative metabolites. Oxidative pathways have not been described for humans, but, if present, tOP in urine may not be the most sensitive biomarker of exposure. Oxidative metabolites are the major urinary metabolites in humans for some other xenobiotics containing long chain alkyl moieties, including the structurally related 4-nonyl phenol (Ye et al. 2007) and phthalate diesters, such as di-isononyl phthalate and di-(2-ethylhexyl) phthalate (Koch and Angerer 2007; Koch et al. 2005). Research is needed to identify and characterize tOP oxidative metabolites that could be used to assess exposure to tOP in humans.

In the last decade, data on the urinary concentrations of BPA in selected populations of various countries have become available (Arakawa et al. 2004; Calafat et al. 2005; Fujimaki et al. 2004; Kim et al. 2003; Liu et al. 2005; Matsumoto et al. 2003; Miyamoto and Kotake 2006; Ouchi and Watanabe 2002; Volkel et al. 2005; Wolff et al. 2007; Yang et al. 2003, 2006). These data suggest that human exposure to BPA is widespread (CERHR 2007 and references therein). However, the urinary concentrations of BPA measured in several population groups show some variation. For example, the median urinary concentration of BPA-glucuronide, detected in all samples collected from 48 female Japanese college students, was 1.2 μg/L (0.77 μg/g creatinine) (Ouchi and Watanabe 2002). By contrast, concentrations of BPA urinary species in seven males and 12 females in Germany were below the LOD of 1.14 μg/L (Volkel et al. 2005), whereas the geometric mean concentration of BPA urinary species in a group of 73 adult Koreans (53% female) was 9.54 μg/L (8.91 μg/g creatinine) (Yang et al. 2003). Although differences in the exposure to BPA may exist geographically, the differences also could be attributed at least partly to differences in timing of urine collection [between 1000 and 1900 hours (Volkel et al. 2005) or before breakfast (Yang et al. 2003)] and analytical detection methods [coulometry (Ouchi and Watanabe 2002), isotope dilution–tandem mass spectrometry (Volkel et al. 2005), or fluorescence spectroscopy (Yang et al. 2003)].

We previously reported the urinary concentrations of free plus conjugated species of BPA in 394 adult participants in the NHANES III callback convenience subsample of about 1,000 persons (Calafat et al. 2005). We detected BPA in 95% of the samples with a geometric mean of 1.3 μg/L (Calafat et al. 2005). The frequency of detection of BPA was similar among the NHANES III callback sub-sample and NHANES 2003–2004. However, the geometric mean of BPA in the NHANES 2003–2004 population was almost double that in the NHANES III callback study population. These differences may be related partly to the small sample size and the not nationally representative nature of the NHANES III callback subsample or to the exclusion of children and adolescents in the NHANES III callback sub-sample, especially because concentrations of BPA are higher in children and teens than in adults in NHANES 2003–2004 (Table 3). Although exposure to BPA (reflected in elevated urinary concentrations of BPA) truly might have increased since the NHANES III 1988–1994 sampling, because of the important differences outlined above, these two data sets are not directly comparable for establishing exposure trends over time. Future NHANES data will be useful in establishing time trends.

BPA geometric mean concentrations were significantly lower in the afternoon collection than in the morning and evening collections; urinary concentrations between the morning and evening sessions were not significantly different. Consumption of food, believed to be a major source of exposure to BPA (Kang et al. 2006; Vandenberg et al. 2007), during the day may result in elevated BPA concentrations in the evening collection samples. In children and adolescents, however, the geometric mean concentrations of BPA did not vary significantly with the time of day of sample collection. These observed variations in the geometric mean concentrations of BPA depending on the time of day of sample collection [Figure 1; Supplemental Material, Table 2 (online at http://www.ehponline.org/members/2007/10753/suppl.pdf)] may reflect variability in exposures as a result of differences in factors such as diet, lifestyle, and use of products containing BPA that may contribute to the observed urinary concentrations of BPA.

Children had significantly higher LSGM concentrations of BPA (4.5 μg/L) than adolescents (3.0 μg/L) and adults (2.5 μg/L), and adolescents also had significantly higher LSGM concentrations than adults (all *p*-values < 0.005; Table 4). Similarly, in a small study conducted in the United States, the median concentrations of BPA urinary species were lower in 23 adults (0.47 μg/L) than in nine 9-year-old girls (2.4 μg/L) (Liu et al. 2005); the BPA concentrations in these nine girls were comparable to the geometric mean concentrations in a group of 90 girls 6–9 years of age [2.0 μg/L (3 μg/g creatinine)] in three locations in the United States (Wolff et al. 2007). Higher urinary concentrations in children than in adults have been reported for other nonpersistent chemicals, such as phthalate metabolites and organophosphate pesticides (CDC 2005). The higher concentrations of BPA in children may be explained by their higher food consumption and air inhalation in relation to their weight than those of adolescents or adults. The differences also could be related to differences in absorption, distribution, metabolism, or excretion of BPA. Nevertheless, our findings highlight the need for additional research to identify the sources and routes of exposure to BPA, especially in children, and the need for epidemiologic studies to target health outcomes related to BPA exposures in children.

Among the NHANES 2003–2004 participants examined, females had significantly higher (*p* = 0.043) LSGM concentrations of BPA than males (Tables 3 and 4). These differences may reflect not only differences in exposure but also differences in pharmacokinetic factors; however, the relevance of these factors is unknown. Furthermore, data are limited regarding the association between BPA concentrations and sex (Kim et al. 2003; Takeuchi and Tsutsumi 2002; Yang et al. 2006). The LSGM concentrations in males and females reported here (Table 3) are similar to those reported among 30 healthy Korean adults (50% men) (Kim et al. 2003). Although the total urinary concentrations of BPA in the Korean men (2.82 ± 0.73 μg/L) and women (2.76 ± 0.54 μg/L) were similar, men had significantly higher (*p* < 0.01) concentrations of BPA-glucuronide than women, and women had significantly higher (*p* < 0.01) concentrations of BPA-sulfate than men (Kim et al. 2003). In addition, no sex-related differences were reported in another study involving 160 Korean adults, 81 of them men (Yang et al. 2006), but urinary concentrations of BPA urinary species both in men and women were considerably higher than concentrations reported for NHANES 2003–2004 participants.

We also observed differences in LSGM concentrations of BPA by race/ethnicity and household income (Tables 3 and 4). Mexican Americans had significantly lower LSGM concentrations of BPA than non-Hispanic whites and non-Hispanic blacks; no statistically significant differences exist between the LSGM concentration of non-Hispanic whites and non-Hispanic blacks. Participants in the low household income category had significantly higher LSGM concentrations than those with the high household income (Tables 3 and 4). These data suggest that race/ethnicity and household income may be associated with factors that affect exposure to BPA.

In summary, we report here the first nationally representative population-based total BPA and tOP concentrations for the U.S. population. These data are a baseline to which concentrations of these chemicals in future sampling of the population can be compared to identify exposure trends. Our data suggest that exposure to BPA in the United States is widespread. We found significant differences in BPA concentrations across selected demographic and income groups. These findings highlight the need for additional research to identify sources and pathways of human exposure to BPA and to evaluate potential health effects that may result from human exposures to BPA.

## Figures and Tables

**Table 1:** Geometric mean and selected percentiles of bisphenol A (BPA) concentrations [μg/L (95% CI)] in urine for the U.S. population ≥ 6 years of age.^a^

Variable	Geometric mean	10th percentile	25th percentile	50th percentile	75th percentile	90th percentile	95th percentile	No.
All	2.6 (2.4–2.9)	0.5 (< LOD–0.6)	1.3 (1.1–1.5)	2.7 (2.4–3.0)	5.4 (5.0–6.1)	10.4 (9.4–12.0)	15.9 (14.4–17.2)	2,517
	2.6 (2.4–2.8)	0.9 (< LOD–1.0)	1.5 (1.3–1.7)	2.5 (2.3–2.8)	4.3 (3.9–4.7)	7.7 (6.6–8.7)	11.2 (9.8–12.4)	2,514
6–11 years	3.6 (2.9–4.3)	0.9 (0.7–1.1)	1.7 (1.2–2.5)	3.7 (2.7–5.0)	6.7 (6.0–8.3)	12.6 (9.5–15.1)	16.0 (11.3–19.0)	314
	4.3 (3.6–5.1)	1.3 (1.2–1.7)	2.7 (1.7–3.3)	4.2 (3.6–5.2)	7.1 (5.8–9.6)	11.8 (9.8–14.8)	15.7 (12.2–23.2)	314
12–19 years	3.7 (3.3–4.2)	0.8 (0.5–1.3)	1.9 (1.6–2.3)	4.2 (3.6–4.6)	7.5 (6.5–9.0)	13.5 (11.8–15.2)	16.5 (15.2–20.9)	715
	2.8 (2.5–3.1)	1.0 (0.9–1.1)	1.7 (1.5–1.9)	2.7 (2.4–3.2)	4.7 (4.2–5.1)	7.8 (6.4–8.9)	11.4 (8.1–14.2)	713
20–59 years	2.6 (2.3–2.9)	0.5 (< LOD–0.6)	1.2 (1.0–1.5)	2.7 (2.4–3.0)	5.2 (4.6–6.0)	9.8 (8.1–12.1)	15.5 (12.5–19.4)	951
	2.4 (2.2–2.6)	0.9 (< LOD–1.0)	1.5 (1.3–1.7)	2.4 (2.2–2.5)	3.9 (3.5–4.3)	6.6 (6.0–7.6)	9.8 (8.5–11.2)	950
≥ 60 years	1.9 (1.6–2.3)	< LOD	0.8 (0.6–1.1)	1.9 (1.6–2.1)	4.1 (3.2–5.2)	8.2 (6.0–11.0)	13.3 (8.9–19.1)	537
	2.3 (1.9–2.6)	< LOD	1.3 (1.1–1.6)	2.2 (1.8–2.6)	3.9 (3.2–4.9)	6.9 (5.7–8.9)	12.1 (8.5–14.0)	537
Female	2.4 (2.1–2.8)	< LOD	1.2 (0.8–1.4)	2.4 (2.2–2.8)	5.0 (4.2–6.2)	10.6 (8.6–12.5)	15.7 (13.5–20.1)	1,288
	2.8 (2.5–3.1)	< LOD	1.7 (1.5–1.8)	2.7 (2.4–2.9)	4.4 (3.8–5.2)	7.9 (6.5–10.2)	12.2 (9.3–18.1)	1,286
Male	2.9 (2.6–3.2)	0.7 (0.5–0.8)	1.4 (1.2–1.6)	3.2 (2.7–3.5)	6.1 (5.4–6.6)	10.4 (9.5–11.4)	16.0 (12.7–17.2)	1,229
	2.4 (2.1–2.6)	0.8 (0.7–1.0)	1.4 (1.2–1.6)	2.3 (2.1–2.7)	4.2 (3.8–4.6)	7.1 (6.4–8.3)	9.9 (9.1–11.7)	1,228
Mexican American	2.6 (2.2–3.1)	0.6 (0.4–0.7)	1.3 (1.0–1.6)	2.6 (2.0–3.1)	5.2 (4.4–6.5)	9.8 (7.3–13.9)	15.4 (10.2–19.7)	613
	2.3 (2.0–2.7)	0.8 (0.5–1.1)	1.4 (1.2–1.6)	2.4 (2.0–2.7)	3.9 (3.2–4.6)	7.1 (5.0–9.0)	10.7 (8.5–14.3)	612
Non-Hispanic black	4.2 (3.7–4.8)	1.2 (0.9–1.3)	2.3 (1.8–2.7)	4.2 (3.7–5.1)	8.1 (7.1–9.7)	14.1 (11.7–16.9)	20.5 (14.9–25.2)	652
	2.9 (2.6–3.3)	0.9 (0.8–1.3)	1.7 (1.4–2.1)	3.0 (2.5–3.3)	4.9 (4.1–6.1)	8.6 (7.5–9.6)	11.8 (10.2–13.3)	651
Non-Hispanic white	2.5 (2.3–2.8)	0.5 (< LOD–0.6)	1.2 (0.9–1.4)	2.7 (2.4–2.8)	5.1 (4.7–5.8)	9.5 (8.3–10.9)	15.1 (12.6–16.7)	1,092
	2.6 (2.4–2.8)	0.9 (< LOD–1.1)	1.5 (1.3–1.7)	2.6 (2.3–2.8)	4.3 (3.9–4.7)	7.6 (6.3–8.9)	11.0 (9.3–12.4)	1,091

CI, confidence interval. Data are from NHANES 2003–2004. Blue lines denote measure in μg/g creatinine.

a Persons not defined by these three race/ethnicity groups were included only in the total population estimate. LOD = 0.4 μg/L. The weighted frequency of detection was 92.6%.

**Table 2:** Selected percentiles of tOP concentrations [μg/L (95% CI)] in urine for the U.S. population ≥ 6 years of age.^a^

Variable	10th percentile	25th percentile	50th percentile	75th percentile	90th percentile	95th percentile	No.
All	< LOD	< LOD	0.3 (< LOD–0.4)	0.9 (0.5–1.2)	1.6 (1.1–2.2)	2.2 (1.6–3.2)	2,517
	< LOD	< LOD	0.3 (< LOD–0.5)	0.9 (0.6–1.3)	1.9 (1.3–2.5)	2.8 (2.0–4.0)	2,514
6–11 years	< LOD	< LOD	0.4 (0.2–0.5)	0.8 (0.5–1.4)	1.6 (1.1–2.1)	2.0 (1.5–2.9)	314
	< LOD	< LOD	0.5 (0.3–0.6)	1.1 (0.7–1.6)	2.0 (1.7–2.2)	2.4 (2.0–6.0)	314
12–19 years	< LOD	< LOD	0.4 (0.2–0.5)	1.1 (0.6–1.5)	1.7 (1.2–2.4)	2.4 (1.6–3.2)	715
	< LOD	< LOD	0.3 (0.2–0.5)	0.7 (0.5–1.2)	1.6 (1.1–2.6)	2.6 (1.5–3.7)	713
20–59 years	< LOD	< LOD	0.2 (< LOD–0.4)	0.7 (0.5–1.2)	1.7 (1.1–2.3)	2.2 (1.5–3.4)	951
	< LOD	< LOD	0.3 (< LOD–0.4)	0.8 (0.5–1.1)	1.6 (1.1–2.7)	2.7 (1.6–3.7)	950
60 years	< LOD	< LOD	0.3 (< LOD–0.4)	1.0 (0.7–1.4)	1.7 (1.1–2.2)	2.4 (1.6–2.9)	537
	< LOD	< LOD	0.4 (< LOD–0.7)	1.2 (0.8–1.7)	2.4 (1.7–3.2)	3.3 (2.3–5.3)	537
Female	< LOD	< LOD	0.2 (< LOD–0.4)	0.9 (0.5–1.2)	1.6 (1.1–2.3)	2.3 (1.5–2.9)	1,288
	< LOD	< LOD	0.4 (< LOD–0.6)	1.0 (0.6–1.4)	2.2 (1.4–3.0)	3.3 (2.4–4.8)	1,286
Male	< LOD	< LOD	0.3 (< LOD–0.4)	0.9 (0.5–1.5)	1.8 (1.2–2.4)	2.2 (1.6–3.2)	1,229
	< LOD	< LOD	0.3 (< LOD–0.4)	0.7 (0.5–1.1)	1.6 (1.1–2.3)	2.4 (1.6–3.3)	1,228
Mexican American	< LOD	< LOD	< LOD	0.5 (0.3–0.6)	0.8 (0.6–1.3)	1.3 (0.8–1.8)	613
	< LOD	< LOD	< LOD	0.4 (0.3–0.6)	0.9 (0.6–1.4)	1.6 (0.9–2.7)	612
Non-Hispanic black	< LOD	< LOD	0.4 (< LOD–0.5)	1.0 (0.7–1.4)	1.9 (1.3–2.6)	2.4 (1.6–3.1)	652
	< LOD	< LOD	0.3 (< LOD–0.4)	0.8 (0.5–1.1)	1.5 (1.1–2.1)	2.3 (1.7–2.8)	651
Non-Hispanic white	< LOD	< LOD	0.3 (< LOD–0.5)	0.9 (0.6–1.5)	1.7 (1.2–2.4)	2.3 (1.6–3.3)	1,092
	< LOD	< LOD	0.4 (< LOD–0.6)	1.0 (0.6–1.4)	2.0 (1.4–3.0)	3.1 (2.2–4.2)	1,091

CI, confidence interval. Data are from NHANES 2003–2004. Blue lines denote measure in μg/g creatinine.

a The geometric mean was not calculated because the overall weighted frequency of detection was < 60% (57.4%). Persons not defined by these three race/ethnicity groups were included only in the total population estimate. LOD = 0.2 μg/L.

**Table 3:** Adjusted LSGM concentrations (95% confidence intervals) of BPA (μg/L) in various demographic groups.

Variable	LSGM (95% CI)
Sex	
Male	2.6 (2.4–2.8)
Female	2.9 (2.6–3.2)
Race/ethnicity	
Mexican American	2.3 (2.0–2.7)
Non-Hispanic white	2.7 (2.5–2.9)
Non-Hispanic black	3.0 (2.6–3.4)
Age (years)	
6–11	4.5 (3.9–5.1)
12–19	3.0 (2.7–3.4)
≥ 20	2.5 (2.3–2.7)
Income	
< $20,000	3.1 (2.7–3.5)
$20,000–$45,000	2.8 (2.6–3.1)
> $45,000	2.5 (2.3–2.7)

**Table 4:** Observed statistical significance values for differences between adjusted LSGM concentrations of BPA for various demographic groups.

Difference	*p*-Value
Females vs. males	0.043
Mexican Americans vs. non-Hispanic whites	0.007
Mexican Americans vs. non-Hispanic blacks	0.006
Non-Hispanic whites vs. non-Hispanic blacks	0.21
Children vs. adolescents	< 0.001
Children vs. adults	< 0.001
Adolescents vs. adults	0.003
< $20,000 vs. $20,000–$45,000	0.17
< $20,000 vs. > $45,000	0.004
$20,000–$45,000 vs. > $45,000	0.088
